# Fat tissue, aging, and cellular senescence

**DOI:** 10.1111/j.1474-9726.2010.00608.x

**Published:** 2010-10

**Authors:** Tamara Tchkonia, Dean E Morbeck, Thomas von Zglinicki, Jan van Deursen, Joseph Lustgarten, Heidi Scrable, Sundeep Khosla, Michael D Jensen, James L Kirkland

**Affiliations:** 1 Robert and Arlene Kogod Center on AgingMayo Clinic, Rochester, MN 55905, USA; 2 Henry Wellcome Biogerontology Laboratory, Institute for Ageing and Health, University of NewcastleNewcastle upon Tyne, UK

**Keywords:** aging, cellular senescence, diabetes, fat tissue, inflammation, obesity, preadipocyte

## Abstract

Fat tissue, frequently the largest organ in humans, is at the nexus of mechanisms involved in longevity and age-related metabolic dysfunction. Fat distribution and function change dramatically throughout life. Obesity is associated with accelerated onset of diseases common in old age, while fat ablation and certain mutations affecting fat increase life span. Fat cells turn over throughout the life span. Fat cell progenitors, preadipocytes, are abundant, closely related to macrophages, and dysdifferentiate in old age, switching into a pro-inflammatory, tissue-remodeling, senescent-like state. Other mesenchymal progenitors also can acquire a pro-inflammatory, adipocyte-like phenotype with aging. We propose a hypothetical model in which cellular stress and preadipocyte overutilization with aging induce cellular senescence, leading to impaired adipogenesis, failure to sequester lipotoxic fatty acids, inflammatory cytokine and chemokine generation, and innate and adaptive immune response activation. These pro-inflammatory processes may amplify each other and have systemic consequences. This model is consistent with recent concepts about cellular senescence as a stress-responsive, adaptive phenotype that develops through multiple stages, including major metabolic and secretory readjustments, which can spread from cell to cell and can occur at any point during life. Senescence could be an alternative cell fate that develops in response to injury or metabolic dysfunction and might occur in nondividing as well as dividing cells. Consistent with this, a senescent-like state can develop in preadipocytes and fat cells from young obese individuals. Senescent, pro-inflammatory cells in fat could have profound clinical consequences because of the large size of the fat organ and its central metabolic role.

## Introduction

Fat tissue is at the nexus of mechanisms and pathways involved in longevity, genesis of age-related diseases, inflammation, and metabolic dysfunction. Major changes in fat distribution and function occur throughout life. In old age, these changes are associated with diabetes, hypertension, cancers, cognitive dysfunction, and atherosclerosis leading to heart attacks and strokes (Guo *et al.*, 1999; Lutz *et al.*, 2008). Excess or dysfunctional fat tissue appears to accelerate onset of multiple age-related diseases, while interventions that delay or limit fat tissue turnover, redistribution, or dysfunction in experimental animals are associated with enhanced healthspan and maximum life span. For example, obesity leads to reduced life span and clinical consequences similar to those common in aging (Ahima, 2009). Conversely, life span is extended: (i) by caloric restriction [which preferentially leads to reduced visceral fat (Barzilai & Gupta, 1999; Masoro, 2006)]; (ii) in fat cell insulin receptor (FIRKO), insulin receptor substrate-1 (IRS-1), and S6 kinase-1 knockout mice [each of which has limited fat development (Bluher *et al.*, 2003; Um *et al.*, 2004; Selman *et al.*, 2008, 2009)]; (iii) in growth hormone receptor knockout (GHRKO) mice [which have reduced IGF-1, delayed increase in the ratio of visceral to subcutaneous fat, and most likely reduced fat cell progenitor turnover (Berryman *et al.*, 2008)]; (iv) with rapamycin treatment [which limits fat tissue development (Chang *et al.*, 2009; Harrison *et al.*, 2009)]; and (v) after surgical removal of visceral fat (Muzumdar *et al.*, 2008). One reason why age-related changes in fat tissue function may entail such profound systemic consequences is that fat is frequently the largest organ in humans. Indeed, it constitutes over half the body in an alarmingly high and increasing number of people [e.g., in women, who have a higher percent body fat than men, with a body mass index (BMI) over 35 kg m^−2^].

Exciting new data are beginning to point to the cell biological and molecular mechanisms that determine how aging impacts fat tissue function and how this, in turn, leads to age-related disease. Lessons from what happens in obesity are especially illuminating. In particular, inflammatory processes linked to cellular senescence in fat tissue could be pivotal. Fat tissue is important in host defense, immunity, injury responses, and production of inflammatory cytokines and chemokines. It is rich in progenitors that can produce pro-inflammatory factors and that are susceptible to cellular senescence. We suggest the possibility that cellular injury responses, activation of innate immunity, accumulation of dysfunctional, dysdifferentiated progenitors [mesenchymal macrophage- and adipocyte-like default (MAD) cells (Kirkland *et al.*, 2002)], and cellular senescence may all be within a spectrum of activated pro-inflammatory fates comprising an alternative differentiation state. Accumulation of senescent cells in fat with aging could be caused by increased generation of these cells owing to a combination of replicative, cytokine-induced, and metabolic stresses as well as reduced removal of senescent cells because of failure of immune cells from older individuals to respond efficiently to chemokines released by senescent cells. In cancer, these chemokines cause the immune system to hone in on senescent cells and the cancer cells around them, resulting in destruction of the cancer cells and removal of the senescent cells (Xue *et al.*, 2007). Furthermore, there are indications that a senescent-like state can occur in nonreplicating, differentiated cells in fat tissue. If true, these points would challenge existing concepts about cellular senescence. To begin to address these hypotheses, relevant findings about aging, obesity, cellular senescence, and inflammation in fat tissue will be considered.

## Fat tissue

### Fat tissue function

In addition to storing energy, fat is important in immune and endocrine function, thermoregulation, mechanical protection, and tissue regeneration. The main role of fat is to store calorically dense fatty acids. These highly reactive, cytotoxic molecules are sequestered as less reactive triglyceride within fat droplets, protecting against systemic lipotoxicity [cytotoxicity owing to fatty acids and lipid metabolites; (Tchkonia *et al.*, 2006a)]. To accommodate wide swings in nutrient availability, fat tissue is capable of rapid, extensive changes in size, especially subcutaneous fat, which is not subject to the anatomic constraints to growth that limit visceral fat. Growth is accomplished through changes in fat cell size or number that vary in magnitude among fat depots.

Adipose tissue is located strategically beneath the skin and around vital organs, where it protects against infection and trauma. Bacterial and fungal infections of fat are uncommon, and metastases are unusual, likely related to the innate and adaptive immune elements in fat tissue, as well as potentially high local fatty acid concentrations that are lethal to pathogens and nonadipose cell types (Tchkonia *et al.*, 2006a). Dysregulated activation of fat tissue immune responses may predispose individuals to the metabolic dysfunction common in both obesity and aging.

Fat tissue produces hormones, including IL-6, angiotensin II, leptin, adiponectin, and IGF-1 (in response to GH), and activates hormones, for example glucocorticoids and sex steroids. It releases paracrine factors in an endocrine-like fashion by developing in target organs and releasing factors that impact their function. For example, fat in muscle regulates muscle glucose homeostasis and insulin responses (Abel *et al.*, 2001). In addition to regional variation in fat tissue endocrine and paracrine factor production, differences in venous drainage contribute to the distinct metabolic effects of different fat depots. For example, omental and some regions of mesenteric fat drains directly into the liver through the portal vein.

Adipose tissue is involved in thermoregulation, both by preventing heat loss through its insulating effects and by generating heat in brown fat. Fat affords mechanical protection by developing at sites of mechanical stress or pressure. It forms a buffer that dissipates pressure over bony prominences, preventing skin breakdown. Fat is rich in mesenchymal progenitors that can give rise to multiple cell types, including fat cells (Cartwright *et al.*, 2007). The multipotent progenitors resident in fat may promote tissue regeneration during wound healing.

### Aging

Fat tissue mass increases through middle age and declines in old age (Visser *et al.*, 2003; Raguso *et al.*, 2006). Fat is redistributed among different fat depots over time, especially during and after middle age, when fat redistributes from subcutaneous to intra-abdominal visceral depots (Meunier *et al.*, 1971; Kotani *et al.*, 1994; Matsuzawa *et al.*, 1995; Kyle *et al.*, 2001; Raguso *et al.*, 2006; Slawik & Vidal-Puig, 2006; Cartwright *et al.*, 2007; Rabkin, 2007; Kuk *et al.*, 2009). Consistent with this, the percent of meal fat stored in subcutaneous depots is lower in older than younger men and women, and abdominal circumference increases by 4.0 cm every 9 years in adult women (Hughes *et al.*, 2004; Koutsari *et al.*, 2009). In old age, fat is redistributed outside fat depots, accumulating in bone marrow, muscle, liver, and other ectopic sites. As in aging, genetic and acquired lipodystrophic syndromes are associated with fat tissue dysfunction, subcutaneous fat loss, increased visceral and ectopic fat, and metabolic syndrome [glucose intolerance, insulin resistance, central obesity, dyslipidemia, and hypertension (Garg & Agarwal, 2009)]. Metabolic syndrome in the elderly, in turn, is associated with increased inflammation, cardiovascular and all-cause mortality, cognitive impairment, and accelerated functional decline (Koster *et al.* 2010; Morley, 2004).

As in humans, mice and rats have fat redistribution and ectopic fat deposition with aging. This is delayed in mouse models with increased maximum life span because of growth hormone and/or insulin-like growth factor-1 (IGF-1) deficiency. While short-term growth hormone exposure decreases visceral fat by enhancing lipolysis, in animals with lifelong excess growth hormone, fat redistribution begins earlier, advances faster, and is associated with decreased life span (Berryman *et al.*, 2004, 2009; Palmer *et al.*, 2009). Removal of visceral fat enhances insulin sensitivity and extends maximum life span in rats (Barzilai *et al.*, 1999; Muzumdar *et al.*, 2008; Huffman & Barzilai, 2009). Conversely, decreased ability of subcutaneous adipocytes to store lipid, as occurs with aging, may contribute to metabolic complications by causing systemic lipotoxicity (Lelliott & Vidal-Puig, 2004; Carley & Severson, 2005; Unger, 2005; Uranga *et al.*, 2005; Tchkonia *et al.*, 2006a; Kuk *et al.*, 2009). Thus, fat redistribution with aging occurs across species and is associated with age-related diseases, lipotoxicity, and reduced longevity, while retention of a high ratio of functioning subcutaneous to visceral fat is associated with enhanced longevity.

Substantial changes in fat tissue metabolic function occur during aging, with declines in insulin, lipolytic, and fatty acid responsiveness (Bertrand *et al.*, 1980; Yu *et al.*, 1982; Kirkland & Dax, 1984; Yki-Jarvinen *et al.*, 1986; Silver *et al.*, 1993; Gregerman, 1994; Kirkland & Dobson, 1997; Kirkland *et al.*, 2002; Das *et al.*, 2004; Tchkonia *et al.*, 2006a). Together with preadipocyte dysfunction, impairments in ability to accumulate or mobilize lipid with aging reduce the dynamic range over which fat cells can store or release energy or respond to excess systemic lipotoxic fatty acids.

Fat tissue cytokines, including tumor necrosis factor-α (TNFα) and interleukin (IL-6), can increase with aging (Morin *et al.*, 1997; Starr *et al.*, 2009). Increased adiponectin, a cytokine that exists in several isoforms and that originates from fat as well as other tissues, has been associated with reduced risk of metabolic syndrome in the elderly (Lim *et al.* 2010; Stenholm *et al.*, 2009). It is higher in centenarians and their offspring than the general population (Atzmon *et al.*, 2008). However, increased adiponectin is associated with increased mortality in the elderly, perhaps related to production by the vascular system in atherosclerosis (Rizza *et al.* 2010; Poehls *et al.*, 2009). More needs to be done to understand whether there are age-related changes in adiponectin production by cells in fat tissue and what the consequences are.

Immune effector abundance appears to change with aging in a fat depot-dependent manner. Macrophage abundance increases in subcutaneous fat with aging in mice (Jerschow *et al.*, 2007). However, macrophage numbers that are already high in intra-abdominal fat of young mice do not increase further with aging (Harris *et al.*, 1999; Jerschow *et al.*, 2007). Little is known about the impact of aging on fat tissue lymphocyte, mast cell, or macrophage abundance in humans.

Brown fat generates heat through uncoupled mitochondrial oxidative phosphorylation. Aging is associated with loss of brown fat as well as brown fat preadipocyte dysfunction in rodents (Gabaldon *et al.*, 1998; McDonald & Horwitz, 1999; Gabaldon *et al.*, 2003). This is delayed by caloric restriction (Valle *et al.*, 2008). Brown fat also decreases with aging in humans, in whom it is interspersed with white fat in the neck and upper chest (Cypess *et al.*, 2009). Decreased brown fat may contribute to thermal dysregulation and energy imbalance.

### Obesity

Obesity has been likened to an accelerated form of fat tissue aging (Ahima, 2009; Minamino *et al.*, 2009; Tchkonia *et al.*, 2009). Obesity causes premature death from many of the same causes as those common in elderly lean individuals: diabetes, heart attacks, strokes, cancer, and dementia (Bjorntorp, 1990; Denke *et al.*, 1993, 1994; Colditz *et al.*, 1995; Lean, 2000). Obesity and aging are both associated with chronic, low-grade inflammation and insulin resistance, increased local and circulating proinflammatory, chemotactic, and procoagulant proteins, and ectopic lipid deposition with lipotoxicity (Hotamisligil & Spiegelman, 1994; Samad *et al.*, 1996, 1997; Vgontzas *et al.*, 1997; Fried *et al.*, 1998; Loffreda *et al.*, 1998; Samad *et al.*, 1998; Ferri *et al.*, 1999; Visser *et al.*, 1999; Perreault & Marette, 2001; De Pergola & Pannacciulli, 2002; Weyer *et al.*, 2002; Sartipy & Loskutoff, 2003; Takahashi *et al.*, 2003; Weisberg *et al.*, 2003; Xu *et al.*, 2003; Curat *et al.*, 2004; Tchkonia *et al.*, 2006a). This is especially true in massive or visceral obesity. As in aging, fat tissue adipogenic transcription factor expression is decreased in obesity, and inflammatory mediators, including TNFα and IL-6, are increased (Nadler *et al.*, 2000; Xu *et al.*, 2002; Weisberg *et al.*, 2003; Xu *et al.*, 2003; Nair *et al.*, 2005; Jerschow *et al.*, 2007). Much more is known about fat tissue dysfunction in obesity than aging. Analysis of processes causing fat tissue dysfunction in obesity could point to mechanisms contributing to metabolic dysfunction with aging and even the aging process itself.

**Fig. 1:**
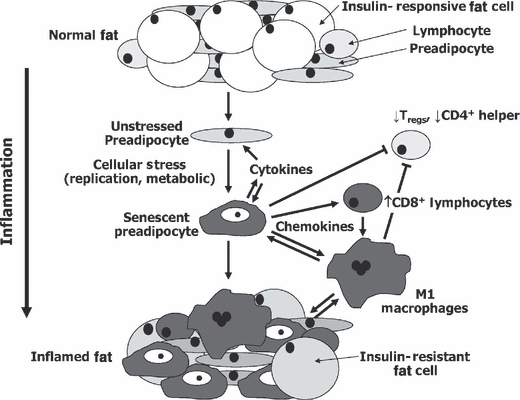
Hypothetical model of the chain of events culminating in fat tissue inflammation in obesity. Preadipocytes and fat tissue endothelial cells may acquire an activated, pro-inflammatory, senescent-like phenotype in response to repeated replication, fatty acids, toxic metabolites, chronically high IGF-1, glucose, or other stimuli (gray = inflamed). Inflammatory cytokines could spread this activated secretory phenotype from cell to cell and block full differentiation of preadipocytes into insulin-responsive fat cells, amplifying the process. Chemokines, cytokines, and ECM modifiers produced by pro-inflammatory cells might activate adaptive immune responses. Shifts from anti- to proinflammatory T-lymphocyte subsets and mast cell infiltration owing to cytokine and lymphokine production, toxic metabolites (including fatty acids and reactive oxygen species), and cytokines released by inflamed preadipocytes and endothelial cells may combine to promote M1 macrophage activation. The inflammatory cytokines could induce systemic effects, further impede adipogenesis, and promote fat cell lipolysis, releasing fatty acids that aggravate the fat tissue pro-inflammatory state and cause systemic lipotoxicity. Similar processes could be involved in age-related fat tissue dysregulation and metabolic dysfunction. Some of these processes appear to vary in extent among fat depots in obesity (Feuerer *et al.*, 2009; Nishimura *et al.*, 2009; Winer *et al.*, 2009) as well as aging (Cartwright *et al.*, 2010).

Events leading to fat tissue inflammation in obesity have been investigated by examining effects of high fat feeding in rodents (Fig. 1). These diets induce fat tissue inflammatory cytokine, chemokine, and extracellular matrix (ECM)-modifying protein production within days to weeks (Xu *et al.*, 2003; Suganami *et al.*, 2005; Nishimura *et al.*, 2009). This is associated with shifts in T-lymphocyte subsets, rather than absolute numbers of T lymphocytes (Feuerer *et al.*, 2009; Nishimura *et al.*, 2009). High fat feeding results in an increased proportion of CD8^+^ effector T lymphocytes, with a progressive increase in cells releasing pro-inflammatory T_H_1 cytokines, relative to CD4^+^ helper cells and T regulatory cells [T_regs_; (Feuerer *et al.*, 2009; Nishimura *et al.*, 2009; Winer *et al.*, 2009)]. This also occurs in humans: CD8A is higher and T_regs_ are reduced in fat tissue from obese compared to lean humans (Feuerer *et al.*, 2009; Nishimura *et al.*, 2009). Fat tissue from obese mice induces CD8^+^ lymphocyte activation and proliferation in coculture (Nishimura *et al.*, 2009), suggesting chemokines produced by fat could be upstream of changes in T-lymphocyte subsets. Fat also becomes infiltrated by mast cells as obesity develops in mice and humans (Liu *et al.*, 2009). Mast cells contribute to production of IL-6, interferon-γ, and metabolic complications of obesity, including insulin resistance and fatty liver.

Following shifts in T-lymphocyte subsets and mast cell accumulation during development of obesity, fat becomes infiltrated by classically activated M1 macrophages (Weisberg *et al.*, 2003; Xu *et al.*, 2003; Kintscher *et al.*, 2008; Liu *et al.*, 2009; Lumeng *et al.*, 2009; Nishimura *et al.*, 2009). This is likely due to chemokines, including interferon-γ [from T lymphocytes, and mast cells (Kintscher *et al.*, 2008; Liu *et al.*, 2009; Sebastian *et al.*, 2009; Winer *et al.*, 2009)], monocyte chemoattractant protein-1 [MCP-1; from preadipocytes and other cell types (Gustafson *et al.*, 2009)], RANTES [from preadipocytes, endothelial cells, and other cell types (Feuerer *et al.*, 2009)], and RARRES2 [from preadipocytes (Kralisch *et al.*, 2009)]. Little MCP-1 or RANTES is produced by fat cells themselves (Fain *et al.* 2009).

The stromal vascular fraction of adipose tissue (comprising preadipocytes, endothelial cells, immune cells, and other cell types) may be the main source of inflammatory cytokines and chemokines produced by fat (Fain *et al.* 2009; Wu *et al.*, 2007; Gustafson *et al.*, 2009). Macrophage infiltration owing to a high fat diet depends more on cells in the stromal vascular fraction of fat tissue than fat cells (Weisberg *et al.*, 2003). Once activated, macrophages release yet more inflammatory cytokines that lead to further production of MCP-1 and other chemokines, inducing further macrophage infiltration and inflammation in a vicious cycle. A central question that has not been fully answered is: what cell types, metabolites, and/or antigens are upstream of the shifts in T-lymphocyte subsets and mast cell accumulation that precede macrophage infiltration?

### Fat tissue distribution in obesity

Different fat depots make distinct contributions to the pro-inflammatory and clinical consequences of obesity and, potentially, aging. Visceral fat enlargement is more strongly associated with ectopic fat deposition, lipotoxicity, and metabolic disease than generalized obesity, especially in old age (Carr *et al.*, 2004; Tchkonia *et al.*, 2006a; Wannamethee *et al.*, 2007; Gustafson *et al.*, 2009; Thomou *et al.*, 2010). Even otherwise lean individuals with relatively more intra- than extra-abdominal fat are at increased risk for diabetes and mortality (Pischon *et al.*, 2008). Removing intra-abdominal fat reduces insulin resistance more profoundly than removing subcutaneous fat from rodents (Barzilai *et al.*, 1999; Weber *et al.*, 2000; Gabriely *et al.*, 2002; Huffman & Barzilai, 2009). Removing large amounts of subcutaneous fat from humans does not improve insulin sensitivity (Klein *et al.*, 2004). Subcutaneous fat expansion in obesity may actually be protective (Kim *et al.*, 2007; Tran *et al.*, 2008). Cytokine and chemokine production by different fat depots varies, with visceral fat being more pro-inflammatory (Samaras *et al.* 2010; Einstein *et al.*, 2005; Tchkonia *et al.*, 2006a; Huffman & Barzilai, 2009; Starr *et al.*, 2009; Thomou *et al.*, 2010). IL-6 levels are higher in visceral than subcutaneous fat in mice, and nutrient excess induces more visceral fat expression of TNFα and plasminogen activator inhibitor-1 (PAI-1), a hemostatic factor associated with atherosclerosis [Einstein *et al.*, 2005; Starr *et al.*, 2009]).

### Is obesity accelerated fat tissue aging?

While obesity is associated with accelerated development of diseases common in old age, mechanisms of fat tissue dysfunction in obesity differ from aging in important ways. Fat cell size is increased in many depots in obesity (Fried & Kral, 1987) and is associated with fat cell death and macrophage infiltration around the dying cells (Cinti *et al.*, 2005). In aging, unlike obesity, this may not contribute substantially to inflammation, because fat cells are generally smaller in old than middle age (Bertrand *et al.*, 1978, 1980). Whole fat tissue gene expression profiles differ in obesity from changes during development (Miard & Picard, 2008). Fat tissue transcripts that change during development (four compared with 12 month old mice) did not correlate well with transcripts affected by obesity (4- month-old obese compared to lean mice). This could be related to differences between changes in fat tissue cellular composition during development from those in obesity. For example, macrophage infiltration in visceral fat from young obese individuals is more impressive than lean old individuals (Weisberg *et al.*, 2003; Xu *et al.*, 2003; Wu *et al.*, 2007).

Whether the basis of fat tissue dysfunction in obesity and aging is the same is more than academic. Clinical consequences of obesity are increasingly close to being amenable to novel interventions based on targeting inflammation and fat tissue cellular composition. For example, antibody-mediated CD8^+^ depletion reduces high fat diet-induced TNFα, IL-6, and M1 macrophage abundance and improves insulin responsiveness in mice (Nishimura *et al.*, 2009). CD3 antibody restores T_regs_, decreases pro-inflammatory M1 relative to anti-inflammatory M2 macrophages, increases antidiabetic IL-10, and reverses insulin resistance for over 4 months despite a high fat diet (Winer *et al.*, 2009). Injection of an IL-2 antibody increases T_regs_ and anti-inflammatory IL10 in abdominal fat and decreases blood glucose in mice on a high fat diet (Feuerer *et al.*, 2009). Transplantation of anti-inflammatory T_H_2 cells into lymphocyte-deficient mice reverses insulin resistance and MCP-1 owing to a high-fat diet (Winer *et al.*, 2009). Reducing mast cells by genetic manipulation or pharmacologic stabilization with disodium chromoglycate reduces hepatic steatosis, circulating inflammatory cytokines and chemokines, angiogenesis, and insulin resistance in obese mice (Liu *et al.*, 2009). Blocking TLR4 in cells originating from bone marrow or ablating CD11c^+^ macrophages reduces insulin resistance in obese mice (Patsouris *et al.*, 2008; Saberi *et al.*, 2009). To the extent that age-related fat tissue dysfunction is similar to obesity, analogous interventions might prevent clinical consequences of age-related fat tissue dysfunction.

## Preadipocytes

### Preadipocyte function

**Fig. 2:**
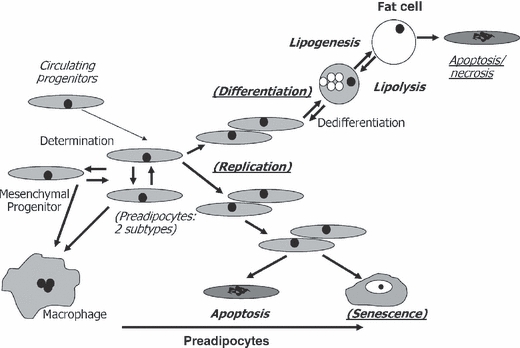
Impact of aging, obesity, anatomic origin, and serial passage on cell dynamic mechanisms of fat tissue turnover. Up to 50% of cells in fat tissue are committed preadipocytes that arise from multipotent, slowly replicating mesenchymal progenitor cells and possibly circulating progenitors (Hong *et al.*, 2005; Crossno *et al.*, 2006). Preadipocyte numbers are maintained by replication. Preadipocytes can reversibly switch into a slowly replicating subtype, can become macrophage-like, and may be able to progress up or down the adipocytic lineage (Cousin *et al.*, 1999; Charriere *et al.*, 2003; Tchkonia *et al.*, 2005; Gustafson *et al.*, 2009). Preadipocytes are depleted by differentiation into fat cells, apoptosis, necrosis, and cellular senescence. Enlargement of fat cells (lipid accumulation) and maintenance of insulin responsiveness are tied to processes initiated during differentiation, including adipogenic transcription factor expression. Fat cells, especially large fat cells, can be removed by a process with features of both apoptosis and necrosis and that can induce inflammation. The balance among these cell dynamic properties determines preadipocyte and fat cell numbers. Cell dynamic processes that vary with aging are in bold, obesity are underlined, anatomic origin in Italics, and serial passage in parentheses. These differences persist for at least 40 population doublings in cloned preadipocytes in the case of anatomic origin, 16 in aging, and 8 in obesity.

Preadipocytes comprise 15–50% of cells in fat, one of the largest progenitor pools in the body (Fig. 2). Preadipocytes replicate in response to mitogens, including IGF-1 (Boney *et al.*, 2001; Sekimoto *et al.*, 2005). They are mainly resident in fat depots, although a small pool of circulating fat cell progenitors exists (Crossno *et al.*, 2006). Preadipocytes, in turn, may arise from or be the same as the multipotent mesenchymal progenitors (also referred to as adipose tissue stem cells) that tend to be aligned along fat tissue blood vessels in a pericyte-like fashion (Tang *et al.*, 2008). Preadipocytes resident in different fat depots are distinct cell subtypes that differ in developmental gene expression and capacities for replication, differentiation, and apoptosis (Yamamoto *et al.* 2010; Kirkland *et al.*, 1990; Tchkonia *et al.*, 2001, 2002, 2006b, 2007b). These differences persist for at least 40 population doublings in strains made by expressing telomerase in single human preadipocytes from different fat depots. Regional differences in preadipocyte clonal capacities for replication and adipogenesis predict subsequent fat tissue growth (Wang *et al.*, 1989).

Preadipocyte metabolic and secretory profiles are distinct from differentiated fat cells and vary among fat depots (Kirkland *et al.*, 1994, 2003; Tchkonia *et al.*, 2007b). Preadipocytes express toll-like receptors and have full innate immune response capability (Lin *et al.*, 2000; Chung *et al.*, 2006; Vitseva *et al.*, 2008). Preadipocytes with activated immune responses likely make a larger contribution than macrophages to age-related fat tissue dysfunction because of their numbers (Xu *et al.*, 2002; Gustafson *et al.*, 2009; Mack *et al.*, 2009). Gene expression profiles of preadipocytes are closer to macrophages than fat cells (Charriere *et al.*, 2003). TNFα and hypoxia induce preadipocytes to release cytokines and chemokines that activate endothelial cells and promote macrophage infiltration (Mack *et al.*, 2009). Treatment of undifferentiated human preadipocytes with TNFα or lipopolysaccharide (LPS) induces CD68, MIP1α, IL-1β, and GM-CSF expression (Gustafson *et al.*, 2009). Activated preadipocytes can even acquire a macrophage-like morphological phenotype (Cousin *et al.*, 1999; Gustafson *et al.*, 2009). Their plasticity and capacity to mount innate immune responses enable preadipocytes to participate in wound repair and defense against infection but also predispose them to contribute to fat tissue inflammation and dysfunction.

The main role of preadipocytes is to give rise to new fat cells. Following initiation of differentiation through signaling pathways activated by fatty acids, IGF-1, glucocorticoids, and other stimuli, a cascade of transcription factors underlies acquisition and maintenance of the fat cell phenotype (Lowell, 1999; Rosen, 2005). The key ‘bottleneck’ in this process is at the level of the adipogenic transcription factors, peroxisome proliferator-activated receptor-γ (PPARγ) and CCAAT/enhancer binding protein-α [C/EBPα; (Lin & Lane, 1994; Hu *et al.*, 1995; Wu *et al.*, 1995; Yeh *et al.*, 1995; Wu *et al.*, 1999; Farmer, 2005)]. PPARγ binds a ligand [e.g., endogenous or dietary lipids or thiazolidinedione (TZD) antidiabetic drugs], heterodimerizes with a ligand-bound retinoid receptor and then induces C/EBPα (Wu *et al.*, 1996; Hamm *et al.*, 2001). C/EBPα, in turn, further increases PPARγ expression (Clarke *et al.*, 1997; Burgess-Beusse *et al.*, 1999). C/EBPα and PPARγ cooperate in regulating downstream adipogenic genes (Hollenberg *et al.*, 1997; El Jack *et al.*, 1999). Sustained activity of both is necessary for development of fully functional, insulin-responsive fat cells and for downregulating the pro-inflammatory proclivities of preadipocytes.

### Aging and preadipocyte function

Extensive changes in preadipocyte function occur with aging [Fig. 2; (Djian *et al.*, 1983; Wang *et al.*, 1989; Kirkland *et al.*, 1990, 1993, 1994, 1997; Kirkland & Dobson, 1997; Kirkland & Hollenberg, 1998; Caserta *et al.*, 2001; Karagiannides *et al.*, 2001; Kirkland *et al.*, 2002; Karagiannides *et al.*, 2006b; Guo *et al.*, 2007; Tchkonia *et al.*, 2007a; Cartwright *et al.*, 2010)]. These include declines in preadipocyte replication (Djian *et al.*, 1983; Kirkland *et al.*, 1990; Kirkland & Hollenberg, 1998; Schipper *et al.*, 2008), decreased adipogenesis (Kirkland *et al.*, 1990, 1993; Karagiannides *et al.*, 2001, 2006b), increased susceptibility to lipotoxicity (Guo *et al.*, 2007), and increased pro-inflammatory cytokine, chemokine, ECM-modifying protease, and stress response element expression (Tchkonia *et al.*, 2007a; Cartwright *et al.*, 2010). These changes progress at different rates and to different extents in preadipocytes from different fat depots (Djian *et al.*, 1983; Kirkland *et al.*, 1990; Schipper *et al.*, 2008; Cartwright *et al.*, 2010). They are inherent: age-dependent declines in replication and differentiation remain evident in most clones derived from single preadipocytes cultured in parallel from animals of different ages for over a month (Djian *et al.*, 1983; Kirkland *et al.*, 1990). However, these changes do not occur uniformly in every preadipocyte: occasional clones derived from old animals replicate and accumulate lipid like the majority of clones from young animals, and some clones from young animals behave more like cells from old animals (Kirkland *et al.*, 1990).

C/EBPα, PPARγ, and their target genes are lower in preadipocytes cultured from older than younger humans and rats following exposure to differentiation medium (Karagiannides *et al.*, 2001; Schipper *et al.*, 2008). PPARγ and C/EBPα are reduced in fat tissue from various species in old age, including primates (Hotta *et al.*, 1999; Karagiannides *et al.*, 2001). These adipogenic transcription factors also decline in serially passaged human preadipocytes exposed to differentiation-inducing medium, with six population doublings being sufficient to detectably impair adipogenesis (Tchkonia *et al.*, 2006b; Noer *et al.*, 2009). Human preadipocyte replicative arrest occurs after approximately 35 population doublings.

The age-related impairment in adipogenesis occurs at a point between the early increase in C/EBPβ transcription and subsequent increases in PPARγ and C/EBPα (Karagiannides *et al.*, 2001). Overexpressing C/EBPα restores capacity for lipid accumulation by preadipocytes from old individuals. Redundant mechanisms impede adipogenesis at this point, including increased expression of C/EBP homologous protein (CHOP) and an alternatively translated, short C/EBPβ isoform, C/EBPβ liver-activating protein (LIP), that lacks the full C/EBPβ-transactivating domain (Karagiannides *et al.*, 2006b; Tchkonia *et al.*, 2007a). Increased binding of CUG triplet repeat-binding protein (CUGBP) to the 5′ region of C/EBPβ mRNA with aging causes LIP to be translated (Karagiannides *et al.*, 2006b). CUGBP activity, LIP, and CHOP are cellular stress responsive and induced by TNFα. Preadipocyte TNFα secretion, in turn, increases with aging (Tchkonia *et al.*, 2007a). Thus, redundant, stress responsive, inherent processes impair adipogenesis with aging.

Decreased adipogenic transcription factors could contribute to age-related declines in fat cell size, capacity to store lipid, and insulin responsiveness [both PPARγ and C/EBPα are required for fat cells to be insulin–responsive; (El Jack *et al.*, 1999)]. These changes occur at different rates in different depots, with subcutaneous depots being particularly affected, potentially contributing to fat redistribution, lipodystrophy, ectopic lipid accumulation, lipotoxicity, and metabolic dysfunction. Even preadipocytes become susceptible to lipotoxicity because of fatty acids in old age, related to reduced expression of adipogenic transcription factors and enzymes required for processing fatty acids into triglycerides (Guo *et al.*, 2007). Fatty acids also induce fat tissue cytokine release (Suganami *et al.*, 2005), further impeding adipogenesis, leading to a downward spiral. While influences extrinsic to fat tissue, including systemic disease and changes in diet, activity, and hormones, likely contribute to fat dysfunction in old age, inherent, age-related changes in preadipocytes set the stage for fat tissue and systemic metabolic dysfunction.

Reduced PPARγ in mouse models is associated with lipodystrophy and reduced life span (Argmann *et al.*, 2009). An increase in maximum life span owing to manipulating PPARγ would need to be demonstrated before concluding definitively that it is involved in progression of aging. On the other hand, gene knock-in replacement of C/EBPα with C/EBPβ results in increased mean and maximum life span together with leanness, resistance to diet-induced obesity, and increased energy expenditure (Chiu *et al.*, 2004). Thus, age-related changes in preadipocyte and fat cell adipogenic transcription factors may contribute not only to morbidity, manipulating them may also prove to delay age-related dysfunction.

### MAD cells

Generation of MAD cells potentially contributes to ectopic lipid accumulation in old age (Kirkland *et al.*, 2002). Preadipocytes are closely related to other mesenchymal progenitors, including osteoblasts, muscle satellite cells, chondroblasts, and macrophages. In old age, muscle satellite cells, osteoblasts, and macrophages can sometimes dysdifferentiate into cells with an incomplete, adipocyte-like phenotype, with lipid accumulation and expression of the fat cell-specific fatty acid-binding protein, aP2, and PPARγ (but insufficient PPARγ for differentiation into fully functional, insulin-responsive fat cells). Failure to express sufficient levels of the transcription factors that direct uncommitted mesenchymal cells into becoming fully functional, specialized cells may contribute to their developing into partially differentiated adipocyte-like cells by default. In muscle, satellite cells from old mice acquire a partial adipocyte phenotype with more lipid accumulation and aP2, C/EBPα, and PPARγ expression than cells from young mice (Taylor-Jones *et al.*, 2002). In bone, osteoblast formation from mesenchymal progenitors is decreased with aging, together with increased adipogenesis (Jilka *et al.*, 1996; Rosen *et al.*, 2009). These changes might contribute to age-related accumulation of fat in bone marrow and muscle as well as osteoporosis.

### Preadipocytes in obesity

Increased fat cell size accounts for increased fat mass in mild obesity, while severe obesity leads to increased numbers of fat cells and preadipocytes, together with increased fat cell turnover because of apoptosis and/or necrosis (Shillabeer *et al.*, 1990; Cinti *et al.*, 2005; Lacasa *et al.*, 2007; Strissel *et al.*, 2007). Preadipocytes are driven to become new fat cells in massive obesity, especially in subcutaneous fat, with preadipocyte replicative history being increased (Cinti *et al.*, 2005; Strissel *et al.*, 2007; Thomou *et al.*, 2010). Up to 10 fold more preadipocytes can be present in very massively obese than lean subjects (Table 1). Additionally, preadipocyte turnover is likely increased, because preadipocytes develop into new fat cells as fat cell number and removal increase (Cinti *et al.*, 2005; Strissel *et al.*, 2007).

**Table 1:** Preadipocyte abundance is increased in obesity

	Nonobese	Obese
Height (m)	1.65 ± 0.02	1.70 ± 0.03
Weight (kg)	70 ± 6	241 ± 6
BMI (m kg^−2^)	25 ± 2	83 ± 2
Estimated body fat (%)	20 ± 2	58 ± 2
Fat tissue (kg)	14 ± 2	139 ± 5
Preadipocytes per g (×10^4^)	45 ± 11	45 ± 6
Preadipocytes/subject (×10^9^)	6.1 ± 2.2	63.1 ± 9.6

Preadipocyte numbers were determined in abdominal subcutaneous fat from five nonobese and 15 massively obese subjects [as in (Kirkland *et al.*, 1994)]. Preadipocytes per g fat tissue differed little between nonobese and obese subjects, as noted by others (van Harmelen *et al.*, 2003). Amount of fat tissue was increased considerably in the obese subjects, resulting in many more preadipocytes/obese subject. The obese subjects had 31 fold more senescent preadipocytes than nonobese subjects (95% confidence limits 13–71), based on preadipocytes/subject and the ratio of SA β–gal^+^ cells in obese/lean fat tissue (=3.04). Mean ± SEM are shown.

BMI, body mass index.

As in chronological aging and after serial passage in culture (both of which are associated with increased preadipocyte replicative histories), in obesity adipogenesis, C/EBPα, PPARγ, and their downstream targets are decreased in preadipocytes and fat tissue (Turkenkopf *et al.*, 1988; Shillabeer *et al.*, 1990; Permana *et al.*, 2004; Nair *et al.*, 2005; Dubois *et al.*, 2006; Tchkonia *et al.*, 2006b; Gustafson *et al.*, 2009). Stromal vascular cells that are aP2^+^ (a downstream target of C/EBPα and PPARγ) are reduced in obese women (Tchoukalova *et al.*, 2007). Impaired adipogenesis in obesity, associated with reduced downregulation of preadipocyte pro-inflammatory genes and restricted capacity to store excess fatty acid as triglyceride, may contribute to fat tissue inflammation, ectopic lipid accumulation, lipotoxicity, and insulin resistance, as occurs in aging (Unger, 2002; Xu *et al.*, 2002; Listenberger *et al.*, 2003; DeFronzo, 2004; Tchkonia *et al.*, 2006a; Kim *et al.*, 2007).

Despite low C/EBPα and PPARγ, fat cell size is usually increased in obesity. Differentiating preadipocytes with low C/EBPα and PPARγ can still accumulate lipid when exposed to fatty acids but are insulin resistant and dysfunctional, consistent with accretion of the large, insulin-resistant, C/EBPα- and PPARγ-deficient fat cells in obesity that are associated with diabetes (Xie *et al.*, 2006; Kim *et al.*, 2007). Restricted capacity to increase fat cell number, with increases in fat cell size occurring instead, is associated with lipotoxicity and elevated diabetes risk (Dubois *et al.*, 2006; Kim *et al.*, 2007). Large fat cells in obesity may have restricted capacity to take up excess fatty acid because of: (i) low adipogenic transcription factor expression and consequently impaired machinery to process fatty acids; (ii) insulin resistance with inhibition of IRS-1 and Glut-4; (iii) increased lipolysis with fatty acid release owing to insulin resistance; (iv) instability from their large size, with increased risk of apoptosis/necrosis; and (v) high fat tissue concentrations of TNFα [which is lipolytic and anti-adipogenic; (Cinti *et al.*, 2005; Xie *et al.*, 2006; Gustafson *et al.*, 2009)]. Fat cell dysfunction in obesity, coupled with reduced ability of preadipocytes to differentiate into fat cells, may contribute to failure to sequester fatty acids, systemic lipotoxicity, and insulin resistance.

## Fat tissue cellular senescence and inflammation

### Cellular senescence

Cellular senescence is considered to be an irreversible block to cell cycle progression in populations of otherwise replication-competent cells (Hayflick & Moorehead, 1961; Narita & Lowe, 2005; Beausejour & Campisi, 2007; Jeyapalan & Sedivy, 2008). Replicative senescence in hyperproliferative states, such as cancer or massive obesity, may constitute a defense against morbidity by removing dysfunctional or excess progenitors from replicating pools of cells (Campisi, 2000, 2004, 2005; Xue *et al.*, 2007). The proportion of arrested cells in a population rises with increasing population doublings, rather than all cells becoming senescent at once (Martin-Ruiz *et al.*, 2004; Passos *et al.*, 2007). This is reflected *in vivo* in particular regions in different organs in old age, with a subset of cells, often less than 10%, expressing p16 and other markers and effectors of senescence (Krishnamurthy *et al.*, 2004; Wang *et al.*, 2009a). In addition to hyperproliferation, cellular senescence or stasis (Stress or Aberrant Signaling-Induced Senescence) can be induced by stresses: telomere shortening, disrupted chromatin, DNA damage, intense mitogenic signals, oncogene activation, metabolic stress, and stress owing to cell culture conditions (Sherr & DePinho, 2000; Narita *et al.*, 2003; Martin-Ruiz *et al.*, 2004; Campisi, 2005). As in replicative senescence, not all cells in a stressed population undergo stasis at the same time, implying that genetic, epigenetic, or paracrine/microenvironmental conditions confer susceptibility to senescence unequally within cell populations. This could be related to somatic drift among the individual cells in tissues (Martin, 2009). Features of cellular senescence include large, flattened cells, enlarged nucleoli, senescence-associated β-galactosidase [SA β-gal] positivity, and other markers and mediators of senescence, such as phospho-Ser15-p53/p21 and p16/hypophosphorylated Rb pathway component expression. Unlike p21, p16 activity appears to increase in nearly all cells as senescence progresses (Jeyapalan & Sedivy, 2008). SA β-gal^+^ cells are increased in hyperproliferative diseases [e.g., cancers, psoriasis, prostatic hypertrophy, atherosclerotic plaques; (Choi *et al.*, 2000; Vasile *et al.*, 2001; Narita & Lowe, 2005; Mimura & Joyce, 2006; Jeyapalan & Sedivy, 2008; Charalambous *et al.*, 2007)].

Cellular senescence takes days to weeks to become fully established, with autocrine biochemical loops involving reactive oxygen species (ROS), IL-6, transforming growth factor-β, and other signals eventually resulting in focal accumulation of heterochromatin (Passos *et al.* 2010; Kuilman *et al.*, 2008; Kuilman & Peeper, 2009; Passos *et al.*, 2009). These heterochromatic foci can be identified by 4′,6-diamidino-2-phenylindole (DAPI) staining and by the activated histones that contribute to DNA repair and stabilization, including γ-phosphorylated histone-2AX [γH2AX; (Wang *et al.*, 2009a)]. In human replicative senescence, heterochromatic foci can be associated with telomeres (telomere-induced foci).

Cellular senescence leads to a senescent secretory phenotype with increased inflammatory cytokines, altered production of ECM-modifying proteases, and production of ROS (Freund *et al.*; Passos *et al.* 2010; Krtolica & Campisi, 2002; Parrinello *et al.*, 2005; Xue *et al.*, 2007; Coppé*et al.*, 2008). Generation of cytokines, chemokines, and ECM modifiers by senescent cells leads to death of cells around them, tissue remodeling, and attraction of immune elements. Although senescent cells are often resistant to apoptosis (Campisi, 2003), activation of the immune system by senescent cells causes removal of nearby cells as well as the senescent cells themselves (Xue *et al.*, 2007). Indeed, activation of innate immunity appears to be required for senescent cells to remove nearby cells. The innate immune response capacity of macrophages appears to be compromised with aging (Sebastian *et al.*, 2009), potentially contributing to senescent cell accumulation in old age.

### Cellular senescence and inflammation in obesity

Obesity and serial passage both entail repeated preadipocyte replication and cellular stress, as well as accumulation of senescent cells, including senescent preadipocytes and endothelial cells (Minamino *et al.*, 2009; Tchkonia *et al.*, 2009). Adipose tissue SA β-gal activity and p53 increase with BMI. Abundance of SA β-gal^+^ cells also increases in fat tissue in diabetes. Interestingly, p53 and p21 are increased in the fat cell fraction from subjects with diabetes (Minamino *et al.*, 2009), suggesting a senescent-like state might occur in differentiated adipocytes, even though these cells are postmitotic and therefore would not fit the usual definition of senescence.

**Fig. 3:**
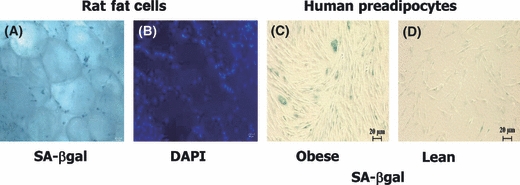
Senescent preadipocytes can accumulate in fat tissue of even young individuals. Freshly isolated, whole perirenal fat tissue isolated from 2-month-old obese male Zucker rats was assayed for senescence-associated β-galactosidase (A; SA β-gal) or stained with DAPI to show nuclei (B; representative of *N* = 3 animals). Preadipocytes cultured from young obese rats had senescent-associated heterochromatic foci. Senescent cells are also increased in high fat-fed mice, express p53 (Minamino *et al.*, 2009), and are less frequent in age-matched *ad libitum* fed controls. Human preadipocytes from an obese young adult subject were SA β-gal positive (C; age 21 years.; body mass index [BMI] 50; representative of six obese subjects), while fewer preadipocytes cultured from a lean subject were senescent (D; age 26 years.; BMI 23; representative of nine lean subjects). Nevertheless, occasional senescent cells were found in preadipocytes cultured from all nine young, lean subjects.

SA β-gal^+^ cells are more numerous in cultures of preadipocytes and endothelial cells isolated from young obese than lean rats and humans [Fig. 3; (Tchkonia *et al.*, 2009)]. Extremely obese subjects can have a burden of over 30-fold more senescent preadipocytes than nonobese subjects (Table 1). These senescent progenitors in fat tissue might initiate the infiltration of immune cells that commonly occurs in obesity, a speculation that merits testing. Immune cells, in turn, could further activate the preadipocyte population into a pro-inflammatory state. Consistent with this possibility, coculture of 3T3-L1 preadipocytes with RAW264 macrophages without direct contact induces a pro-inflammatory state with increased TNFα expression in the preadipocytes (Suganami *et al.*, 2005). Neutralizing anti-TNFα antibody prevents this.

A high burden of senescent cells in obesity could have substantial clinical impact because: (i) senescent cells restricted to a single tissue can have widespread systemic effects (Keyes *et al.*, 2006); (ii) many of the pro-inflammatory cytokines and chemokines released by these cells are associated with development of diabetes and metabolic disease; and (iii) fat is frequently the largest organ in humans. Consistent with this, upregulating p53 in fat cells and macrophages induces senescence and increases insulin resistance and inflammatory cytokines in mouse fat (Minamino *et al.*, 2009). Conversely, expressing dominant-negative p53 in fat cells and macrophages confers protection against the insulin resistance and increased fat tissue cytokines, macrophage infiltration, and SA β-gal activity caused by high fat diets (Minamino *et al.*, 2009). Thus, cellular senescence owing to high p53 and the resulting pro-inflammatory secretory phenotype could contribute to morbidity associated with obesity.

Diabetes itself is associated with cellular senescence in fat tissue. Fat tissue from diabetic humans has increased SA β-gal activity and p53, TNFα, and MCP-1 expression (Minamino *et al.*, 2009). Other fat tissue disorders, such as lipodystrophy in patients with human immunodeficiency virus (HIV) infection treated with certain antiretroviral drugs, cause fat redistribution, diabetes, and metabolic syndrome. The antiretrovirals stavudine and zidovudine cause senescence, with SA β-gal positivity, senescent morphology, and increased p16 and p21, in cultured human fibroblasts and 3T3-F442A murine preadipocytes (Caron *et al.*, 2008). Fat tissue from patients with HIV who are on these drugs can contain senescent cells. Induction of senescence in 3T3-F442A preadipocytes, which are an immortal cell line, indicates these agents can induce preadipocyte senescence independently of replicative history. Cyclo-oxygenase-2 decreases in parallel with induction of senescence by these antiretrovirals, suggesting an association with oxidative stress. Consistent with this, exposure of human preadipocytes to H_2_O_2_ causes increased levels of p53, TNFα, and MCP-1 (Minamino *et al.*, 2009).

### Fat tissue inflammation and cellular senescence with aging

As in obesity, aging is frequently associated with increased fat tissue and circulating pro-inflammatory cytokines, including TNFα and IL-6 (Morin *et al.*, 1997; Starr *et al.*, 2009). Increased pro-inflammatory cytokine release by preadipocytes activates adjacent cells into a pro-inflammatory state: TNFα exposure increases preadipocyte TNFα mRNA (Mack *et al.*, 2009). Although TNFα and hypoxia also increase preadipocyte cytokines that promote endothelial cell-monocyte adhesion and macrophage infiltration (Mack *et al.*, 2009), macrophages do not appear to be a major source of pro-inflammatory cytokines with aging in fat tissue (Wu *et al.*, 2007). Capacity of macrophages to be activated into a pro-inflammatory state by chemokines and cytokines generally declines with aging (Sebastian *et al.*, 2009). While macrophages increase in particular fat depots with aging, these increases are less impressive than in obesity, and macrophage numbers do not increase at all in some fat depots with aging (Harris *et al.*, 1999; Jerschow *et al.*, 2007). This is consistent with the possibility that fat cells and preadipocytes could be the main sources of the increased fat tissue inflammatory cytokines and chemokines with aging. Preadipocytes from old rats release more TNFα than from young rats, with extent of TNFα release by preadipocytes from old animals being similar to macrophages (Tchkonia *et al.*, 2007a). IL-6 expression is also higher in preadipocytes from old than younger rats (Cartwright *et al.*, 2010). Preadipocytes from old rats and the cytokines they produce can impede adipogenesis in nearby fat cells, potentially contributing to age-related lipodystrophy and fat redistribution (Tchkonia *et al.*, 2007a; Gustafson *et al.*, 2009).

Age-related increases in fat tissue inflammatory cytokine and chemokine expression vary among fat depots (Starr *et al.*, 2009; Cartwright *et al.*, 2010). Basal IL-6, complement factor 1q, and MMP3 and MMP12 increase with aging in extraperitoneal but not intraperitoneal rat preadipocytes (Cartwright *et al.*, 2010). Increases in IL-6 caused by treatment with LPS are sixfold higher in visceral (epididymal) and 33-fold higher in interscapular subcutaneous fat from old (27 months) than younger (6 months) mice (Starr *et al.*, 2009). Thus, the extent of the age-related increase in IL-6 response is 5- to 10-fold greater in subcutaneous than visceral fat, suggesting that age-related changes in fat tissue function are more extensive in subcutaneous than visceral fat. Consistent with this, preadipocyte pro-inflammatory cytokine, chemokine, and ECM modifier production is greater in extra- than intra-peritoneal fat in old age (Cartwright *et al.*, 2010). These differences among depots in the trajectory of age-related preadipocyte dysfunction may contribute to disproportional loss of subcutaneous fat.

Age-related changes in fat tissue inflammatory profiles resemble those in obesity, in which senescent preadipocytes and endothelial cells accumulate, as well as the senescent secretory phenotype reported in skin fibroblasts and other cell types *in vitro* [Table 2; (Krtolica & Campisi, 2002; Parrinello *et al.*, 2005; Xue *et al.*, 2007; Coppé*et al.*, 2008; Minamino *et al.*, 2009; Tchkonia *et al.*, 2009; Cartwright *et al.*, 2010)]. SA β-gal activity and p16 reactivity are increased in fat tissue of mice with accelerated aging phenotypes owing to hypomorphism of the *Bubr1* gene (Baker *et al.*, 2006) and after several generations of telomerase deficiency (Minamino *et al.*, 2009). Importantly, p16^Ink4a^ ablation prevents accumulation of senescent cells in BubR1 hypomorphic mice, implicating p16^Ink4a^ in establishing the senescent phenotype in this model (Baker *et al.*, 2008). Together, these findings suggest senescent cells could accumulate in fat tissue with chronological aging and that these cells might contribute to age-related fat tissue inflammation and dysfunction.

**Table 2:** Parallels among preadipocyte and fat tissue changes in obesity, chronological aging, and after repeated replication of cultured preadipocytes and fibroblasts

Property	Obesity	Repeated replication	Aging
Dysdifferentiation	√	√*	√
↑ Inflammation	√	√	√
↑ TNFα, IL6, MMPs, PAI-1	√	√†	√‡
Altered progenitor shape	√	√	√
Insulin resistance	√		√
↑ Senescence associated β-gal	√§	√	√
↓β oxidation, PGC-1α	√¶		√
↑ Stathmin-like-2 (Stmn-2)	√		√‡

Cell dynamic and molecular mechanisms underlying fat tissue dysfunction in obesity in younger individuals are strikingly similar to aging. Similarities between changes in human preadipocyte and fibroblast function after serial passage *in vitro* to those in preadipocytes from obese or old subjects further support this (Tchkonia *et al.*, 2006b, 2009). Thus, obesity, in some respects, resembles an accelerated form of fat tissue aging, potentially involving fat cell progenitor hyperplasia and cellular stress.

* In (Tchkonia *et al.*, 2006b; Noer *et al.*, 2009).

† In (Mu & Higgins, 1995).

‡ (Cartwright *et al.*, 2010).

§ In (Minamino *et al.*, 2009; Tchkonia *et al.*, 2009).

¶ In (Semple *et al.*, 2004). Other references appear in the text.

## Hypothetical model and potential implications

Cellular senescence could be pivotal in the impact of fat tissue on systemic metabolism and healthspan. Cellular senescence, arguably a normally adaptive response to injury or infection, could instead become a root cause of inflammation, failure to sequester fatty acids, and dysfunction both in fat tissue and systemically during aging and in obesity (Fig. 1). In fat, extensive progenitor turnover, high fatty acid levels, toxic metabolites, prolonged IGF-1 exposure, and other mitogens could initiate senescence. Senescence might then spread from cell to cell, involving differentiated fat cells as well as preadipocytes and endothelial cells. Cytokines and chemokines produced by senescent cells appear to be capable of activating adaptive and innate immune responses that could spread cellular senescence locally and systemically. ECM-modifying proteases might expose fat tissue autoantigens or generate neoantigens, further exacerbating the process. Failure to remove senescent cells may contribute to their accumulation, both because of age-related macrophage dysfunction and effects of ECM-modifying proteases on receptors and other proteins required for optimal immune clearance. If this hypothetical model is valid, senescent cells and their products would be a logical target for therapeutic intervention in age- and obesity-related metabolic disease.

This speculative model and recent findings about fat tissue cellular senescence and inflammation prompt several questions about cellular senescence (Table S1). Among these are the following: (i) Is cellular senescence effectively an alternative form of differentiation? (ii) Can a senescent-like state develop in terminally differentiated cells? (iii) Can senescence occur at any stage during life? (iv) Does senescence spread from cell to cell in fat tissue *in vivo*? (v) Does failure of the immune system to remove senescent cells contribute to their accumulation in old age? and (vi) Is cellular senescence really at the root of age- and obesity-related fat tissue inflammation and metabolic dysfunction? As discussed later, suggestive evidence supports affirmative answers to some of these questions, but more work is required to address them definitively.

Is cellular senescence effectively an alternative form of differentiation? Cellular senescence can be viewed as a response to cellular stress (Ben-Porath & Weinberg, 2004). In preadipocytes, cellular senescence, the age-associated MAD state, the pro-inflammatory secretory state activated by cytokines, LPS, fatty acids, ROS, ceramide, cellular stress pathways, or other signals, and the macrophage-like phenotype that preadipocytes can assume may all be related. In addition to preadipocytes, other types of progenitors appear to be able to assume a state very much like M1 activated macrophages, based on their gene expression profiles (Charriere *et al.*, 2006). Acquisition of this pro-inflammatory state appears to involve extra- and intracellular signals that integrate to activate: (i) a chain of signaling molecules; (ii) an orchestrated cascade of transcription factors; (iii) banks of pro-inflammatory cytokines, chemokines, and ECM-modifying proteases; (iv) mechanisms that shut down replication; and (v) mechanisms that prevent usual differentiation. Perhaps senescence is an alternative cell fate that has much in common with pro-inflammatory stress-activated states.

Can a senescent-like state develop in terminally differentiated cells? Terminally differentiated fat cells might be able to acquire a senescent-like state in both aging and obesity (Wu *et al.*, 2007; Minamino *et al.*, 2009). Perhaps the view that a cellular senescent-like state can only occur in dividing cells needs reconsideration.

Can senescence occur at any stage during life? If our hypothetical model is correct, cellular senescence should be inducible at any phase in life. Even at early passage, some cells in cultured human skin fibroblast strains express markers of senescence (Martin-Ruiz *et al.*, 2004). The proportion of these cells increases with passaging or following imposition of cellular stress at early passage (Passos *et al.*, 2007). With respect to fat, senescent cells can be found in fat tissue and preadipocytes from juvenile animals and young adult humans with obesity (Fig. 3). The answer to this question appears to be yes.

Does cellular senescence spread from cell to cell? Cultured human cells and mouse cells *in vivo* that have DNA double-strand breaks and γH2AX induced by radiation can induce DNA damage responses and γH2AX foci in nearby cells (Sokolov *et al.*, 2007). Cytokines secreted by activated preadipocytes, macrophages, and endothelial cells can induce chemokine and cytokine expression in bystander cells in a pattern like that of the senescent secretory phenotype (Suganami *et al.*, 2005; Mack *et al.*, 2009). TNFα induces cellular senescence in preadipocytes, endothelial progenitor cells, and skin fibroblasts (Keyes *et al.*, 2006; Tchkonia *et al.*, 2009; Zhang *et al.*, 2009). Thus, it seems senescence can spread locally from cell to cell. It will be interesting to test whether fat tissue cellular senescence induced by obesity leads to generation of senescent cells elsewhere, such as in the brain. Indeed, high fat feeding induces cellular senescence in aortic endothelium, a process that is mediated by Akt and mTOR and inhibited by rapamycin (Wang *et al.*, 2009b). Whether aortic endothelial senescence results directly from increased circulating lipids owing to the high fat diet, hormones affected by the diet (e.g., insulin or IGF-1), cytokines released by senescent cells in fat or elsewhere, or a combination of mechanisms remains to be determined.

Does failure of the immune system to remove senescent cells contribute to their accumulation in old age? Macrophage function generally declines with aging (Sebastian *et al.*, 2009). Age-related alterations in the stromal microenvironment, including cytokine imbalance, could further impede macrophage function (Stout & Suttles, 2005), possibly augmenting senescent cell accumulation. This question needs to be addressed specifically in fat tissue macrophages, because macrophage properties are highly tissue dependent (Stout & Suttles, 2005; Sebastian *et al.*, 2009).

Is cellular senescence at the root of age- and obesity-related fat tissue inflammation and metabolic dysfunction? The potentially central role of preadipocytes in genesis of fat tissue inflammation and metabolic dysfunction has not received much attention. Preadipocyte cellular stress can result from repeated replication, hypoxia, ROS, chronic effects of free fatty acids or other lipids such as ceramide, hyperglycemia, or other metabolic signals. These appear to activate innate immune responses in preadipocytes, causing further cytokine and chemokine generation, potentially inducing spread of activation of pro-inflammatory responses to nearby preadipocytes and other cell types and attraction of immune cells. The preadipocyte pro-inflammatory phenotype might impede protection against lipotoxicity, contributing to systemic consequences in aging similar to those in lipodystrophies and obesity. The pathways and processes culminating in this generation of activated or senescent preadipocytes and fat cells represent potential new targets for intervention.

It seems a senescent-like, pro-inflammatory fate can be acquired in response to intra- or extracellular danger signals, inflammation, infection, excessive replication, or toxic metabolites. This spectrum of cell fates may prevent differentiation along pathways that are not desirable in the context of damage. In the case of fat tissue, this could involve preventing development of new fat cells or enlargement of existing ones in favor of acquiring cells capable of facilitating repair. Perhaps terminally differentiated cells are able to enter a senescent secretory state related to the molecular pathways that initiate senescence in dividing cell types. Studies in fat tissue are beginning to suggest that cellular senescence could be an alternative cell fate involving activation of pro-inflammatory responses owing to intra- or extracellular injury signals at any stage of life.

## Supporting Information

**Table S1** Research questions regarding cellular senescence and fat tissue

As a service to our authors and readers, this journal provides supporting information supplied by the authors. Such materials are peer-reviewed and may be re-organized for online delivery, but are not copy-edited or typeset. Technical support issues arising from supporting information (other than missing files) should be addressed to the authors.
