# Supplementary material for: Fat tissue, aging, and cellular senescence
Source: Aging Cell. 2010 Oct;9(5):667–84. doi: 10.1111/j.1474-9726.2010.00608.x (PMC2941545; doi:10.1111/j.1474-9726.2010.00608.x)
Supplement: Supplementary file 1 [file acel0009-0667-SD1.doc]

**Supplemental Table 1** Research questions regarding cellular senescence and fat tissue

**Senescent cell properties**

Can cellular senescence occur in post-mitotic as well as proliferating cell types?

Do senescent cells activate both innate and adaptive immunity?

Is cellular senescence the same as the pro-inflammatory state that macrophages and many types of progenitors are capable of entering into?

Does the preadipocyte senescent secretory phenotype vary among preadipocytes from different fat depots?

Is the senescent secretory phenotype different in progenitors from old than younger individuals?

Are MAD cells senescent?

Does failure of the immune system to remove senescent cells contribute to accumulation with aging?

Do immune cells from old animals have reduced ability to be attracted and activated by cellular senescence?

Do senescent progenitors from old animals elicit less inflammation than senescent progenitors from young animals?

Is cellular senescence less effective in stopping cancer in older subjects because it does not elicit as strong a pro-inflammatory immune response?

Can senescent cells be shielded from immune surveillance because of the proteases they produce?

Do proteases from senescent cells induce autoimmunity by exposing autoantigens or creating neoantigens?

**Signals inducing senescence**

What metabolic signals associated with obesity cause senescence (e.g., fatty acids, glucose, ceramide, ROS)?

Do these signals contribute to cellular senescence in tissues besides fat (e.g., heart, liver, brain)?

Does IGF-1 or GH promote fat tissue senescent cell accumulation?

**Conditions predisposing to fat tissue cellular senescence**

Could the genetic factors that predispose to different patterns of fat distribution also cause differences in senescent cell accumulation among depots?

Do lipodystrophies predispose to senescence in fat tissue?

Does “creeping” fat have increased abundance of senescent cells (fat tissue expands at sites of injury, e.g., around the bowel in inflammatory bowel disease and around the heart in congestive heart failure. This “creeping” fat has activated inflammatory pathways [Karagiannid*es et a*l. 2006a; Gro*ss et a*l. 2009])?

Does the senescent secretory phenotype in fat cause senescence in other tissues?

**Preventing, blocking, or reversing senescence**

Is senescence a reversible process *in vivo*?

If cellular senescence has a reversible component, does capacity of cells to revert from senescence change with age?

Does caloric restriction delay accumulation of senescent cells in fat (food restriction delays many changes associated with aging, increases maximum lifespan, reduces markers of inflammation and macrophage infiltration in fat tissue (Higa*mi et a*l. 2006), and delays age-related increases in p16 [Krishnamurt*hy et a*l. 2004])?

Does weight loss decrease senescent cell abundance?

How long does this take?

Does rapamycin block cellular senescence in fat tissue and its clinical consequences in aging, obesity, or lipodystrophies?

**Consequences and implications of senescence**

Does removing senescent cells or blocking their effects restore function?

Does fat tissue senescent cell accumulation have systemic consequences over and above those due to fat tissue metabolic disturbance?

Are senescent cells an impediment to successful organ or stem cell transplantation (senescent cell cytokines, chemokines, and ECM modifiers could make the microenvironment unfavorable for engraftment as well as potentially affecting adaptive immunity, promoting graft versus host disease and rejection)?
